# The genome sequence of the Water Carpet, *Lampropteryx suffumata *(Denis & Schiffermiiller, 1775)

**DOI:** 10.12688/wellcomeopenres.19654.1

**Published:** 2023-07-12

**Authors:** Douglas Boyes, Peter W.H. Holland, University of Oxford and Wytham Woods Genome Acquisition Lab, Darwin Tree of Life Barcoding collective, Wellcome Sanger Institute Tree of Life programme, Wellcome Sanger Institute Scientific Operations: DNA Pipelines collective, Tree of Life Core Informatics collective, Darwin Tree of Life Consortium

**Affiliations:** 1 UK Centre for Ecology & Hydrology, Wallingford, England, UK; 2 University of Oxford, Oxford, England, UK

**Keywords:** Lampropteryx suffumata, Water Carpet, genome sequence, chromosomal, Lepidoptera

## Abstract

We present a genome assembly from an individual male *Lampropteryx suffumata* (the Water Carpet; Arthropoda; Insecta; Lepidoptera; Geometridae). The genome sequence is 581.6 megabases in span. Most of the assembly is scaffolded into 31 chromosomal pseudomolecules, including the Z sex chromosome. The mitochondrial genome has also been assembled and is 16.48 kilobases in length. Gene annotation of this assembly on Ensembl identified 18,663 protein coding genes.

## Species taxonomy

Eukaryota; Metazoa; Eumetazoa; Bilateria; Protostomia; Ecdysozoa; Panarthropoda; Arthropoda; Mandibulata; Pancrustacea; Hexapoda; Insecta; Dicondylia; Pterygota; Neoptera; Endopterygota; Amphiesmenoptera; Lepidoptera; Glossata; Neolepidoptera; Heteroneura; Ditrysia; Obtectomera; Geometroidea; Geometridae; Larentiinae; *Lampropteryx*; *Lampropteryx suffumata* (Denis & Schiffermiiller, 1775) (NCBI:txid934945).

## Background

Many species of insect are found widely across the Palaearctic realm from Europe to Asia, but relatively few have distributions that also extend to North America. Some exceptions include highly migratory species and those transported by human activity. A land connection existed until around five million years ago, the Bering land bridge, and until the land connection was lost there was some dispersal of species in each direction ( Gladenkov *et al.*, 2002; Jiang *et al.*, 2019). The Water Carpet *Lampropteryx suffumata* is a moth in the family Geometridae now known to exist on both sides of the Bering Strait. In Eurasia, the species is found commonly in northern and central Europe with the largest numbers of records from Britain, Scandinavia and Austria; there are scattered records ranging from Ireland and France in the west across to the far east of Russia, including Khabarovsk Krai and the Kamchatka peninsula, and on the Japanese island of Hokkaido ( Beljaev & Vasilenko, 2002; Beljaev *et al.*, 2022). In 2000 eight specimens of *L. suffumata* were recorded from Alaska, and in 2008 several Canadian specimens in museum collections were retrospectively identified as *L. suffumata* by DNA barcoding, including a historical specimen dating from 1919 ( Choi, 2000; Dewaard *et al.*, 2008). There is no suggestion that these North American individuals were accidentally introduced. Hence, the longitudinal range of *L. suffumata* extends from the Dingle Peninsula, Ireland, eastward across Eurasia and the Bering Strait to reach Alberta, Canada.

Like most ‘carpet’ moths, named for their resemblance to the intricate patterns on woven carpets, the Water Carpet rests with its wings flat against the surface in a delta shape. The forewings are silvery-grey with a deeply indented brown cross-band outlined in white. In Britain and Ireland, the moth is on the wing from March to May, with the emergence time having moved earlier in the year since the 1970s ( Randle *et al.*, 2019). The species is most commonly encountered in damp woodland, moorland and fens, where the larvae feed on cleavers ( *Galium aparine*) and other *Galium* species; the pupal stage overwinters ( South, 1961; Waring *et al.*, 2017).

The complete genome sequence of *Lampropteryx suffumata* was determined as part of the Darwin Tree of Life project. The assembled genome will facilitate studies into the biogeography and population genetics of this widespread species and contribute to the growing set of resources for studying insect ecology and evolution.

## Genome sequence report

**Figure 1.**
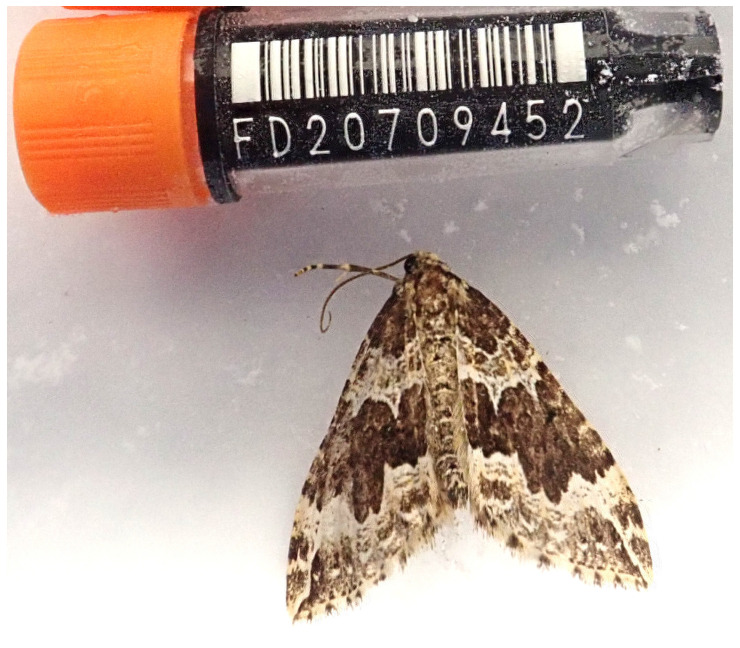
Photograph of the *Lampropteryx suffumata* (ilLamSuff1) specimen used for genome sequencing.

The genome was sequenced from one male *Lampropteryx suffumata* ( Figure 1) collected from Wytham Woods, Oxfordshire, UK (51.77, –1.34). A total of 39-fold coverage in Pacific Biosciences single-molecule HiFi long was generated. Primary assembly contigs were scaffolded with chromosome conformation Hi-C data. Manual assembly curation corrected 31 missing joins or mis-joins and removed 16 haplotypic duplications, reducing the assembly length by 1.99 % and the scaffold number by 11.86%, and increasing the scaffold N50 by 1.22%.

**Figure 2.**
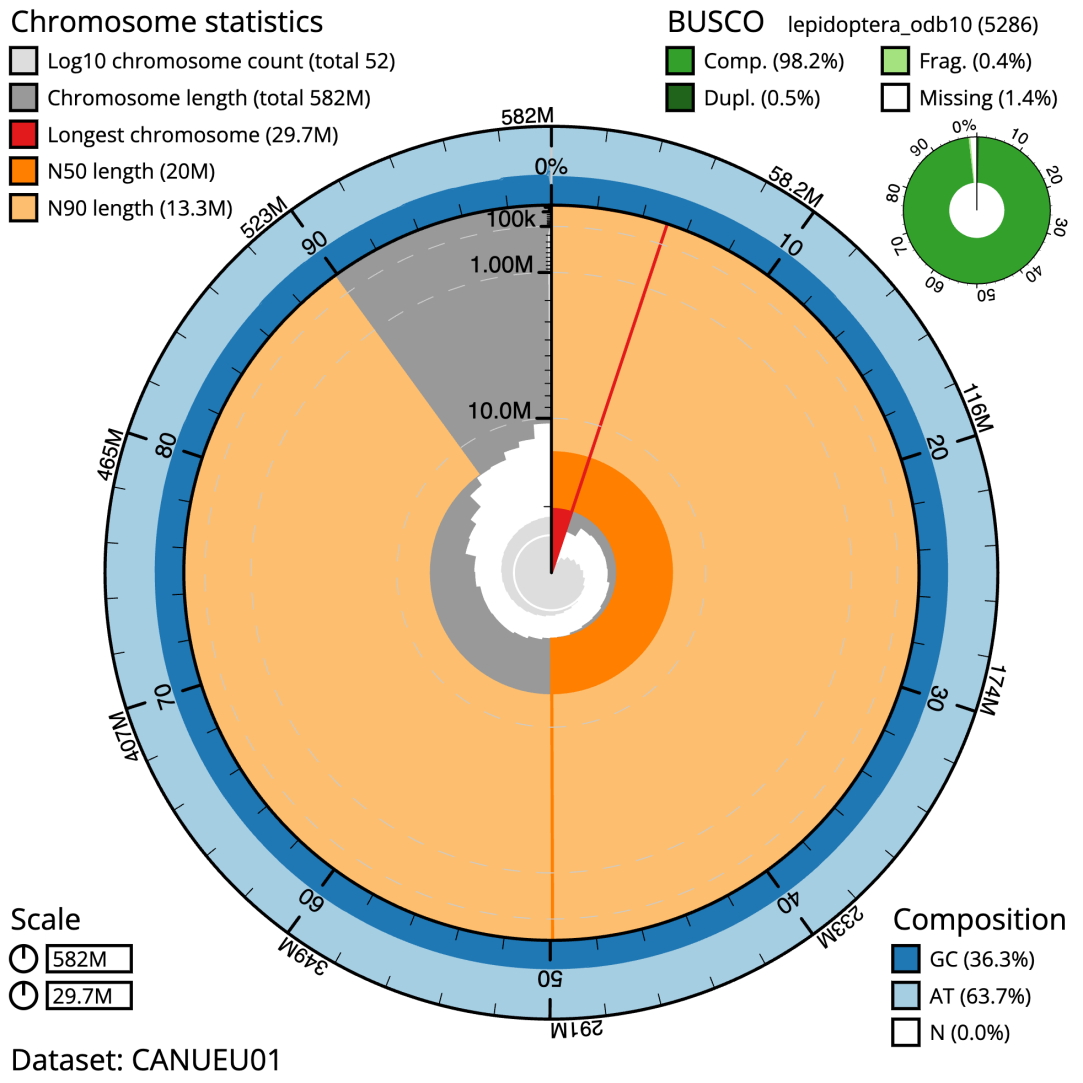
Genome assembly of *Lampropteryx suffumata*, ilLamSuff1.1: metrics. The BlobToolKit Snailplot shows N50 metrics and BUSCO gene completeness. The main plot is divided into 1,000 size-ordered bins around the circumference with each bin representing 0.1% of the 581,655,136 bp assembly. The distribution of scaffold lengths is shown in dark grey with the plot radius scaled to the longest scaffold present in the assembly (29,738,398 bp, shown in red). Orange and pale-orange arcs show the N50 and N90 scaffold lengths (19,956,036 and 13,321,282 bp), respectively. The pale grey spiral shows the cumulative scaffold count on a log scale with white scale lines showing successive orders of magnitude. The blue and pale-blue area around the outside of the plot shows the distribution of GC, AT and N percentages in the same bins as the inner plot. A summary of complete, fragmented, duplicated and missing BUSCO genes in the lepidoptera_odb10 set is shown in the top right. An interactive version of this figure is available at https://blobtoolkit.genomehubs.org/view/Lampropteryxsuffumata/dataset/CANUEU01/snail.

The final assembly has a total length of 581.6 Mb in 51 sequence scaffolds with a scaffold N50 of 20.0 Mb ( Table 1). Most (99.81%) of the assembly sequence was assigned to 31 chromosomal-level scaffolds, representing 30 autosomes and the Z sex chromosome. Chromosome-scale scaffolds confirmed by the Hi-C data are named in order of size ( Figure 2– Figure 5; Table 2). The Z chromosome was identified based on synteny with *Eulithis prunata* (GCA_918843925.1) ( Boyes & Holland, 2023). While not fully phased, the assembly deposited is of one haplotype. Contigs corresponding to the second haplotype have also been deposited. The mitochondrial genome was also assembled and can be found as a contig within the multifasta file of the genome submission.

**Table 1.** Genome data for *Lampropteryx suffumata*, ilLamSuff1.1.

Project accession data		
Assembly identifier	ilLamSuff1.1	
Species	*Lampropteryx suffumata*	
Specimen	ilLamSuff1	
NCBI taxonomy ID	934945	
BioProject	PRJEB58348	
BioSample ID	SAMEA10107026	
Isolate information	ilLamSuff1, male: thorax (DNA sequencing), head (Hi-C scaffolding), abdomen (RNA sequencing)	
Assembly metrics *		*Benchmark*
Consensus quality (QV)	64.7	*≥ 50*
*k*-mer completeness	100%	*≥ 95%*
BUSCO **	C:98.2%[S:97.7%,D:0.5%], F:0.4%,M:1.4%,n:5,286	*C ≥ 95%*
Percentage of assembly mapped to chromosomes	99.81%	*≥ 95%*
Sex chromosomes	Z chromosome	*localised homologous* * pairs*
Organelles	Mitochondrial genome assembled	*complete single* * alleles*
Raw data accessions		
PacificBiosciences SEQUEL II	ERR10677858	
Hi-C Illumina	ERR10684086	
PolyA RNA-Seq Illumina	ERR11242515	
Genome assembly		
Assembly accession	GCA_948098915.1	
*Accession of alternate* * haplotype*	GCA_948098925.1	
Span (Mb)	581.6	
Number of contigs	122	
Contig N50 length (Mb)	9.5	
Number of scaffolds	51	
Scaffold N50 length (Mb)	20.0	
Longest scaffold (Mb)	29.7	
Genome annotation		
Number of protein-coding genes	18,663	
Number of gene transcripts	18,828	

* Assembly metric benchmarks are adapted from column VGP-2020 of “Table 1: Proposed standards and metrics for defining genome assembly quality” from ( Rhie *et al.*, 2021). ** BUSCO scores based on the lepidoptera_odb10 BUSCO set using v5.3.2. C = complete [S = single copy, D = duplicated], F = fragmented, M = missing, n = number of orthologues in comparison. A full set of BUSCO scores is available at https://blobtoolkit.genomehubs.org/view/Lampropteryx suffumata/dataset/CANUEU01/busco.

**Figure 3.**
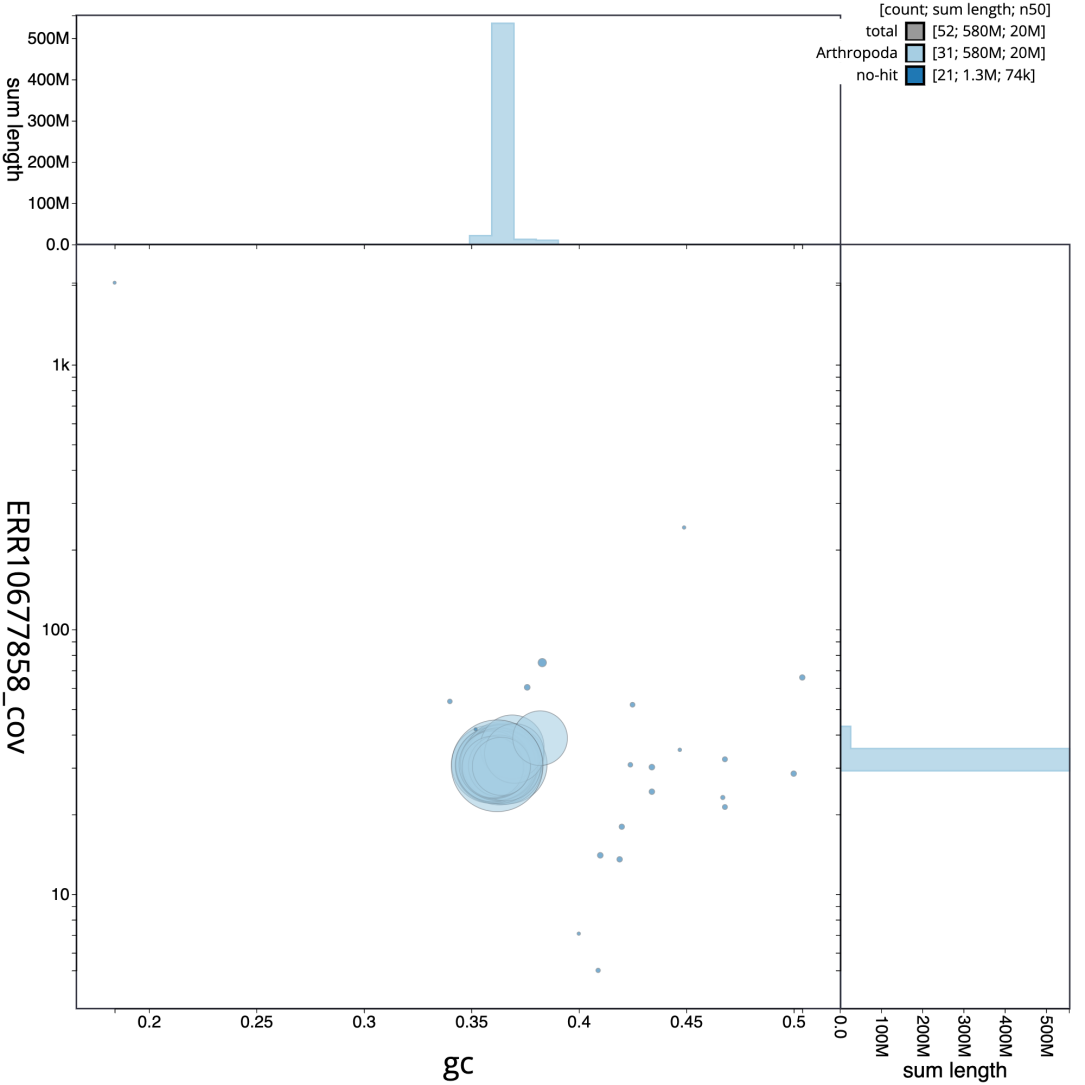
Genome assembly of *Lampropteryx suffumata*, ilLamSuff1.1: BlobToolKit GC-coverage plot. Scaffolds are coloured by phylum. Circles are sized in proportion to scaffold length. Histograms show the distribution of scaffold length sum along each axis. An interactive version of this figure is available at https://blobtoolkit.genomehubs.org/view/Lampropteryxsuffumata/dataset/CANUEU01/blob.

**Figure 4.**
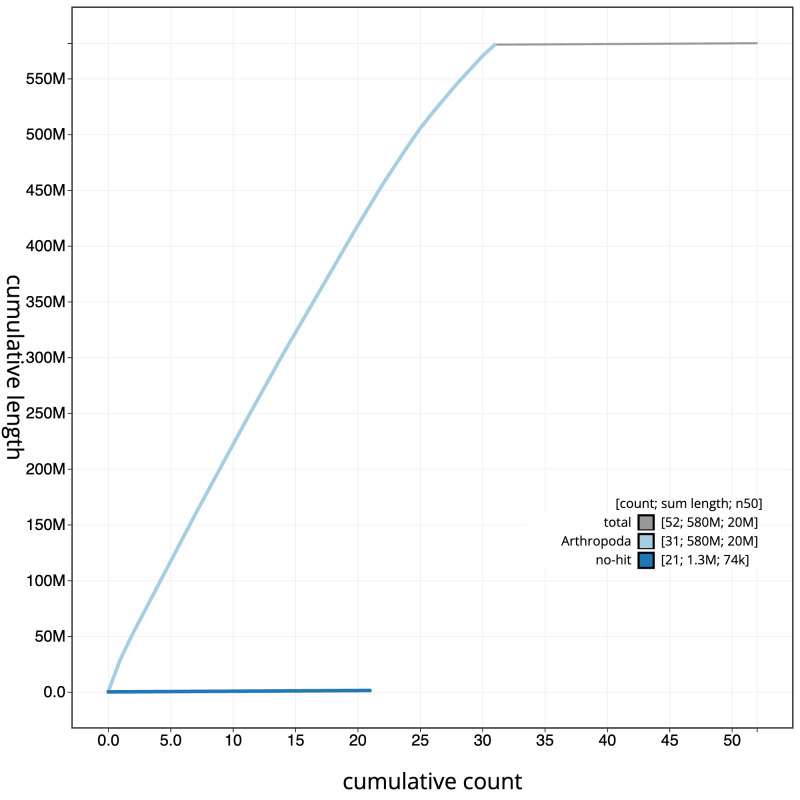
Genome assembly of *Lampropteryx suffumata*, ilLamSuff1.1: BlobToolKit cumulative sequence plot. The grey line shows cumulative length for all scaffolds. Coloured lines show cumulative lengths of scaffolds assigned to each phylum using the buscogenes taxrule. An interactive version of this figure is available at https://blobtoolkit.genomehubs.org/view/Lampropteryxsuffumata/dataset/CANUEU01/cumulative.

**Figure 5.**
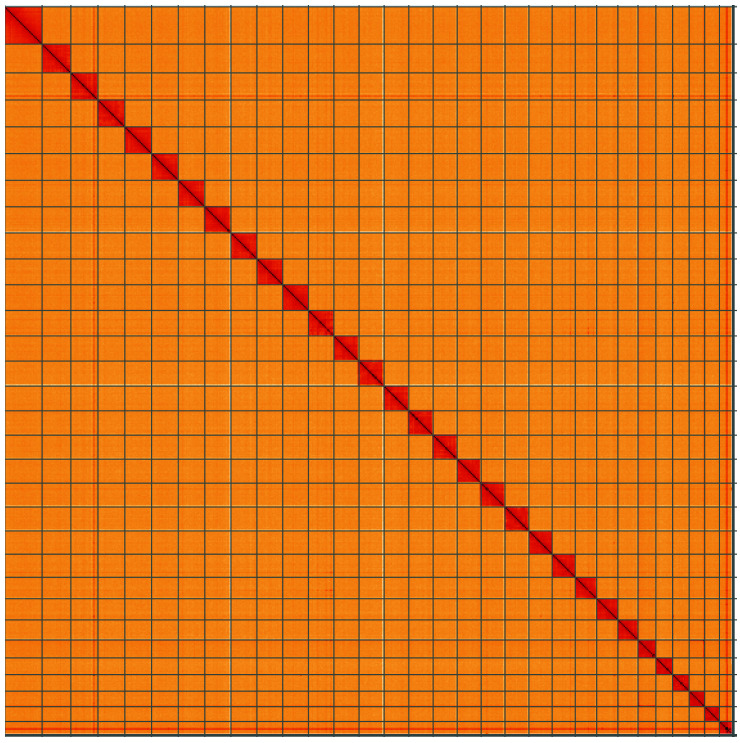
Genome assembly of *Lampropteryx suffumata*, ilLamSuff1.1: Hi-C contact map of the ilLamSuff1.1 assembly, visualised using HiGlass. Chromosomes are shown in order of size from left to right and top to bottom. An interactive version of this figure may be viewed at https://genome-note-higlass.tol.sanger.ac.uk/l/?d=TwZBJG1cSRCC_If8XAVIzA.

**Table 2.** Chromosomal pseudomolecules in the genome assembly of *Lampropteryx suffumata*, ilLamSuff1.

INSDC accession	Chromosome	Length (Mb)	GC%
OX402545.1	1	22.97	36.5
OX402546.1	2	21.6	36.5
OX402547.1	3	21.41	36.0
OX402548.1	4	21.33	36.5
OX402549.1	5	21.26	36.5
OX402550.1	6	21.19	36.0
OX402551.1	7	20.83	36.0
OX402552.1	8	20.76	36.5
OX402553.1	9	20.58	36.0
OX402554.1	10	20.5	36.5
OX402555.1	11	20.31	36.5
OX402556.1	12	20.08	36.5
OX402557.1	13	19.96	36.0
OX402558.1	14	19.72	36.0
OX402559.1	15	19.39	36.0
OX402560.1	16	19.15	36.5
OX402561.1	17	19.13	36.0
OX402562.1	18	19.06	36.5
OX402563.1	19	19.04	36.5
OX402564.1	20	18.56	36.0
OX402565.1	21	18.43	36.5
OX402566.1	22	17.08	36.5
OX402567.1	23	16.89	36.0
OX402568.1	24	16.04	36.0
OX402569.1	25	14.27	37.0
OX402570.1	26	13.32	36.5
OX402571.1	27	13.21	36.0
OX402572.1	28	12.39	37.0
OX402573.1	29	11.81	36.5
OX402574.1	30	10.39	38.0
OX402544.1	Z	29.74	36.0
OX402575.1	MT	0.02	18.5

The estimated Quality Value (QV) of the final assembly is 64.7 with *k*-mer completeness of 100%, and the assembly has a BUSCO v5.3.2 completeness of 98.2% (single = 97.7%, duplicated = 0.5%), using the lepidoptera_odb10 reference set ( *n* = 5,286).

Metadata for specimens, spectral estimates, sequencing runs, contaminants and pre-curation assembly statistics can be found at https://links.tol.sanger.ac.uk/species/934945.

## Genome annotation report

The *Lampropteryx suffumata* genome assembly (GCA_948098915.1) was annotated using the Ensembl rapid annotation pipeline ( Table 1; https://rapid.ensembl.org/Lampropteryx_suffumata_GCA_948098915.1/Info/Index). The resulting annotation includes 18,828 transcribed mRNAs from 18,663 protein-coding genes.

## Methods

### Sample acquisition and nucleic acid extraction

The specimen used for genome sequencing was a male *Lampropteryx suffumata* (specimen ID Ox001102, ilLamSuff1) was collected from Wytham Woods, Oxfordshire (biological vice-county Berkshire), UK (latitude 51.77, longitude –1.34) on 2021-03-31 using a light trap. The specimen was collected and identified by Douglas Boyes (University of Oxford), and was snap-frozen on dry ice.

The specimen was prepared for DNA extraction at the Tree of Life laboratory, Wellcome Sanger Institute (WSI). The ilLamSuff1 sample was weighed and dissected on dry ice with tissue set aside for Hi-C sequencing. Thorax tissue was disrupted using a Nippi Powermasher fitted with a BioMasher pestle. DNA was extracted at the WSI Scientific Operations core using the Qiagen MagAttract HMW DNA kit, according to the manufacturer’s instructions.

RNA was extracted from abdomen tissue of ilLamSuff1 in the Tree of Life Laboratory at the WSI using TRIzol, according to the manufacturer’s instructions. RNA was then eluted in 50 μl RNAse-free water and its concentration assessed using a Nanodrop spectrophotometer and Qubit Fluorometer using the Qubit RNA Broad-Range (BR) Assay kit. Analysis of the integrity of the RNA was done using Agilent RNA 6000 Pico Kit and Eukaryotic Total RNA assay.

### Sequencing

Pacific Biosciences HiFi circular consensus DNA sequencing libraries were constructed according to the manufacturers’ instructions. Poly(A) RNA-Seq libraries were constructed using the NEB Ultra II RNA Library Prep kit. DNA and RNA sequencing were performed by the Scientific Operations core at the WSI on Pacific Biosciences SEQUEL II (HiFi) and Illumina NovaSeq 6000 (RNA-Seq) instruments. Hi-C data were also generated from head tissue of ilLamSuff1 using the Arima2 kit and sequenced on the Illumina NovaSeq 6000 instrument.

### Genome assembly, curation and evaluation

Assembly was carried out with Hifiasm ( Cheng *et al.*, 2021) and haplotypic duplication was identified and removed with purge_dups ( Guan *et al.*, 2020). The assembly was then scaffolded with Hi-C data ( Rao *et al.*, 2014) using YaHS ( Zhou *et al.*, 2023). The assembly was checked for contamination and corrected as described previously ( Howe *et al.*, 2021). Manual curation was performed using HiGlass ( Kerpedjiev *et al.*, 2018) and Pretext ( Harry, 2022). The mitochondrial genome was assembled using MitoHiFi ( Uliano-Silva *et al.*, 2022), which runs MitoFinder ( Allio *et al.*, 2020) or MITOS ( Bernt *et al.*, 2013) and uses these annotations to select the final mitochondrial contig and to ensure the general quality of the sequence.

A Hi-C map for the final assembly was produced using bwa-mem2 ( Vasimuddin *et al.*, 2019) in the Cooler file format ( Abdennur & Mirny, 2020). To assess the assembly metrics, the *k*-mer completeness and QV consensus quality values were calculated in Merqury ( Rhie *et al.*, 2020). This work was done using Nextflow ( Di Tommaso *et al.*, 2017) DSL2 pipelines “sanger-tol/readmapping” ( Surana *et al.*, 2023a) and “sanger-tol/genomenote” ( Surana *et al.*, 2023b). The genome was analysed within the BlobToolKit environment ( Challis *et al.*, 2020) and BUSCO scores ( Manni *et al.*, 2021; Simão *et al.*, 2015) were calculated.

Table 3 contains a list of relevant software tool versions and sources.

**Table 3.** Software tools: versions and sources.

Software tool	Version	Source
BlobToolKit	4.1.5	https://github.com/blobtoolkit/ blobtoolkit
BUSCO	5.3.2	https://gitlab.com/ezlab/busco
Hifiasm	0.16.1- r375	https://github.com/chhylp123/ hifiasm
HiGlass	1.11.6	https://github.com/higlass/higlass
Merqury	MerquryFK	https://github.com/thegenemyers/ MERQURY.FK
MitoHiFi	2	https://github.com/marcelauliano/ MitoHiFi
PretextView	0.2	https://github.com/wtsi-hpag/ PretextView
purge_dups	1.2.3	https://github.com/dfguan/purge_ dups
sanger-tol/ genomenote	v1.0	https://github.com/sanger-tol/ genomenote
sanger-tol/ readmapping	1.1.0	https://github.com/sanger-tol/ readmapping/tree/1.1.0
YaHS	1.2a	https://github.com/c-zhou/yahs

### Genome annotation

The BRAKER2 pipeline ( Brůna *et al.*, 2021) was used in the default protein mode to generate annotation for the *Lampropteryx suffumata* assembly (GCA_948098915.1) in Ensembl Rapid Release.

### Wellcome Sanger Institute – Legal and Governance

The materials that have contributed to this genome note have been supplied by a Darwin Tree of Life Partner. The submission of materials by a Darwin Tree of Life Partner is subject to the **‘Darwin Tree of Life Project Sampling Code of Practice’**, which can be found in full on the Darwin Tree of Life website here. By agreeing with and signing up to the Sampling Code of Practice, the Darwin Tree of Life Partner agrees they will meet the legal and ethical requirements and standards set out within this document in respect of all samples acquired for, and supplied to, the Darwin Tree of Life Project.

Further, the Wellcome Sanger Institute employs a process whereby due diligence is carried out proportionate to the nature of the materials themselves, and the circumstances under which they have been/are to be collected and provided for use. The purpose of this is to address and mitigate any potential legal and/or ethical implications of receipt and use of the materials as part of the research project, and to ensure that in doing so we align with best practice wherever possible. The overarching areas of consideration are:

•   Ethical review of provenance and sourcing of the material

•   Legality of collection, transfer and use (national and international) 

Each transfer of samples is further undertaken according to a Research Collaboration Agreement or Material Transfer Agreement entered into by the Darwin Tree of Life Partner, Genome Research Limited (operating as the Wellcome Sanger Institute), and in some circumstances other Darwin Tree of Life collaborators.

## Data Availability

European Nucleotide Archive: *Lampropteryx suffumata* (water carpet). Accession number PRJEB58348; https://identifiers.org/ena.embl/PRJEB58348. ( Wellcome Sanger Institute, 2023) The genome sequence is released openly for reuse. The *Lampropteryx suffumata* genome sequencing initiative is part of the Darwin Tree of Life (DToL) project. All raw sequence data and the assembly have been deposited in INSDC databases. Raw data and assembly accession identifiers are reported in Table 1.

## Author information

Members of the University of Oxford and Wytham Woods Genome Acquisition Lab are listed here: https://doi.org/10.5281/zenodo.4789928.

Members of the Darwin Tree of Life Barcoding collective are listed here: https://doi.org/10.5281/zenodo.4893703.

Members of the Wellcome Sanger Institute Tree of Life programme are listed here: https://doi.org/10.5281/zenodo.4783585.

Members of Wellcome Sanger Institute Scientific Operations: DNA Pipelines collective are listed here: https://doi.org/10.5281/zenodo.4790455.

Members of the Tree of Life Core Informatics collective are listed here: https://doi.org/10.5281/zenodo.5013541.

Members of the Darwin Tree of Life Consortium are listed here: https://doi.org/10.5281/zenodo.4783558.
