# Supplementary material for: The genome sequence of the Water Carpet, Lampropteryx suffumata (Denis & Schiffermiiller, 1775)
Source: Wellcome Open Res. 2026 Mar 9;8:304. Originally published 2023 Jul 12. [Version 2] doi: 10.12688/wellcomeopenres.19654.2 (PMC13080333; doi:10.12688/wellcomeopenres.19654.2)
Supplement: Peer review history

# Reviewer report: Reviewer response for version 2

**DOI:** 10.21956/wellcomeopenres.28659.r151917

**Published:** 2026-04-14

**Authors:** Wai Lok So

**Affiliations:** 1 The Hang Seng University of Hong Kong (Ringgold ID: 272025), Hong Kong, Hong Kong

The manuscript presents a high-quality genome assembly of the moth, *Lampropteryx suffumata,* as a part of the Darwin Tree of Life initiative. The assembly methods are technically sound with excellent assembly scores. The authors have made sufficient amendments and improvements regarding the comments provided by the earlier reviewers. The resulting resource would be beneficial to future lepidopteran ecological and evolutionary studies.

 I have no major concerns with the manuscript.

Are sufficient details of methods and materials provided to allow replication by others?

Yes

Is the rationale for creating the dataset(s) clearly described?

Yes

Are the datasets clearly presented in a useable and accessible format?

Yes

Are the protocols appropriate and is the work technically sound?

Yes

Reviewer Expertise:

evolution, genomics, arthropods, myriapods

I confirm that I have read this submission and believe that I have an appropriate level of expertise to confirm that it is of an acceptable scientific standard.

# Reviewer report: Reviewer response for version 1

**DOI:** 10.21956/wellcomeopenres.21771.r77250

**Published:** 2024-07-04

**Authors:** Xiaolei Huang

**Affiliations:** 1 Fujian Agriculture and Forestry University, Fuzhou, Fujian, China

The article reports a genome assembly of the carpet moth Lampropteryx suffumata, which is a widespread species with longitudinal range across the Eurasia and the North America. The genome sequence data will facilitate phylogenetics, biogeography and population genetic studies.

 As part of the Darwin Tree of Life project, this Data Note article follows a standardized format, and presents sufficient details of research materials and analysis procedures. I think the article is well prepared. My only minor suggestion is maybe the authors can provide more details such as parameter configurations of specific tools using in some key analyses, if the authors also think this is necessary.

Are sufficient details of methods and materials provided to allow replication by others?

Yes

Is the rationale for creating the dataset(s) clearly described?

Yes

Are the datasets clearly presented in a useable and accessible format?

Yes

Are the protocols appropriate and is the work technically sound?

Yes

Reviewer Expertise:

insect ecology and evolution

I confirm that I have read this submission and believe that I have an appropriate level of expertise to confirm that it is of an acceptable scientific standard.

# Reviewer report: Reviewer response for version 1

**DOI:** 10.21956/wellcomeopenres.21771.r83936

**Published:** 2024-06-06

**Authors:** Christopher B. Cunningham

**Affiliations:** 1 University of Georgia, Athens, Georgia, USA

The project continues to produce quality resources for the insect genomics community. The methods are sound and relatively well documented. The article needs a few more details to be reasonably reproducible.

 Abstract. There is no justification within the abstract, just results. At least a sentence that this was part of a large survey of insects is needed. And one sentence for its possible value.

 Keywords. Not present.  

 Background. There is little true motivation for this study in the introduction. Nothing wrong is said, but the reader has little idea why the work was done. Almost all the details of its life history are irrelevant to the reader.

 Figure 2/3/4/5 titles. These should be informative and tell the reader what you would like them to understand about the data; e.g., Fig 3. Little contamination was found in the final genome assembly.

 Figure 4. has very little information and can easily be summarized as a sentence in the results. The same is true of Fig 5

 All parameter setting if they were different from default of each piece of bioinformatic software needs to be specified. Just put a sentence to begin that everything was default unless specified otherwise. What assessment was done to ensure that default was adequate?

 A reasonable, likely slightly high, number of genes is predicted. However, the reader has very little understanding of how these were evaluated beyond their initial generation. What QC beyond an automated annotation was done? One annotation run with default parameters should come with a strong disclaimer that any further work on specific gene families requires QC on the part of the user because the current annotation is a guide only.

             What is the BUSCO percentage of the predicted gene set? That is an absolute minimum for a reader to be able to assess the minimum quality of the annotation. How many orthogroups with other insects, etc?

 Table 3 is of little to no value and contains much duplicated information. Just add the version numbers of software used in line at the appropriate places in the methods section.

 Is the Ethics statement needed? It just says the provider of the sample should meet some agreed upon standard, but does not actually say that they did in this case. Either drop the statement or actually say the standard was met for this sample. This is not a paper about the standards of collection.

 Add something to the report that contextualize the research for the reader. Is this species the first of a taxon to be assembled, is it an outgroup to some established model species, is it now possible to investigate some interesting aspect of the species biology, etc? This does not to be extensive or highly directed, but there is currently no justification of this work outside of the Data Availability Statement. Even Data Note should contain rationale for the work.

Are sufficient details of methods and materials provided to allow replication by others?

Partly

Is the rationale for creating the dataset(s) clearly described?

Yes

Are the datasets clearly presented in a useable and accessible format?

Yes

Are the protocols appropriate and is the work technically sound?

Yes

Reviewer Expertise:

Behavior, Reproduction, Genetics, Genomics, Epigenetics, Insects

I confirm that I have read this submission and believe that I have an appropriate level of expertise to confirm that it is of an acceptable scientific standard, however I have significant reservations, as outlined above.

# Response

**Published:** 2026-02-05

**Authors:** Tree of Life Team Sanger

**Affiliations:** Wellcome Trust Sanger Institute, UK

Thank you for reviewing this data note. Several of the comments raise broader points about the scope and purpose of data notes. While we have addressed points where clarification was appropriate, the manuscript intentionally follows the established data note format, which is designed to describe the generation, structure and quality of a dataset rather than to present biological interpretation or comparative analyses. **Abstract: **We have added a sentence stating that this genome assembly was generated as part of a large-scale eukaryote sequencing effort within the Darwin Tree of Life project. **Keywords: **These are provided in article metadata and appear in the PDF version. This is determined by the journal layout rather than the manuscript content. **Background and rationale: **The Background section provides a concise rationale for sequencing this species in the context of the Darwin Tree of Life programme. This programme aims to generate high-quality reference genomes across taxonomic breadth rather than to address a single hypothesis for each species. We have reviewed available sequence data and added a sentence to the background, which supports the importance of the genome presented here. **Figures 2–5: **These figures present standard quality assessments used across genome data notes, allowing consistency and comparability across studies. Their purpose is to summarise assembly properties and quality metrics rather than to test hypotheses. **Parameter settings and defaults: **All analyses were run using standard pipelines with default parameters unless otherwise stated. We have added a link to the GitHub repository for Tree of Life production code. **Genome annotation and quality control: **Genome annotation was produced by an external annotation team using established automated workflows. We link to these resources to ensure accessibility for users, but the annotation was not generated or curated by the authors of this manuscript. **Table 3: **(Now Table 4.) is retained as a consolidated reference for software tools and repositories used. Listing version numbers inline throughout the Methods would substantially reduce readability. We note that reviewer preferences on this point vary, and the current presentation aligns with other published data notes. **Ethics statement: **The Ethics statement is a mandatory requirement for our institute and reflects compliance with agreed standards for sample collection.
